# The *Coprinopsis cinerea* Tup1 homologue Cag1 is required for gill formation during fruiting body morphogenesis

**DOI:** 10.1242/bio.021246

**Published:** 2016-11-04

**Authors:** Ryo Masuda, Naoki Iguchi, Kooki Tukuta, Takahiro Nagoshi, Kazuki Kemuriyama, Hajime Muraguchi

**Affiliations:** Department of Biotechnology, Faculty of Bioresource Sciences, Akita Prefectural University, Akita 010-0195, Japan

**Keywords:** Cap, *Coprinopsis cinerea*, Fruiting, Gill trama, Pileus, Tup1

## Abstract

The pileus (cap) of the fruiting body in homobasidiomycete fungi bears the hymenium, a layer of cells that includes the basidia where nuclear fusion, meiosis and sporulation occur. *Coprinopsis cinerea* is a model system for studying fruiting body development. The hymenium of *C. cinerea* forms at the surface of the gills in the pileus. In a previous study, we identified a mutation called *cap-growthless1-1* (*cag1-1*) that blocks gill formation, which yields primordia that never mature. In this study, we found that the *cag1* gene encodes a homologue of *Saccharomyces cerevisiae* Tup1. The *C. cinerea* genome contains another Tup1 homologue gene called *Cc.tupA*. Reciprocal tagging of Cag1 and Cc.TupA with green and red fluorescent proteins revealed that the relative ratios of the amounts of the two Tup1 paralogues varied among tissues. Compared with Cc.TupA, Cag1 was preferentially expressed in the gill trama tissue cells, suggesting that the function of Cag1 is required for gill trama tissue differentiation and maintenance. Yeast two-hybrid analysis and co-localisation of Cag1 and Cc.TupA suggested that Cag1 interacts with Cc.TupA in the nuclei of certain cells.

## Abstract (precis)

**Summary:** Cag1, a Tup1 homologue in *Coprinopsis cinerea*, is required for gill formation in fruiting body development and is highly expressed in the central region of gills.

## INTRODUCTION

The basidiomycete fungus *Coprinopsis cinerea* produces a highly differentiated multicellular structure as the fruiting body (Kües, 2000; Muraguchi and Kamada, 1998). Fruiting body formation begins with an aggregation of hyphae, which produces hyphal knots measuring about 0.2 mm or less in diameter. In the hyphal knots, the cells divide rapidly and differentiate into a compact core comprising highly branched short cells and a layer of veil cells covering the core (van der Valk and Marchant, 1978). After the differentiation of the primordial shaft tissue, the rudimentary pileus differentiates at the upper region of the primordial shaft to form a tiny fruiting body primordium (Muraguchi and Kamada, 1998). The fruiting body primordium gradually enlarges and matures under appropriate light conditions, such as a 12 h light:12 h dark cycle (Kamada et al., 1978; Terashima et al., 2005).

**Fig. 1.**
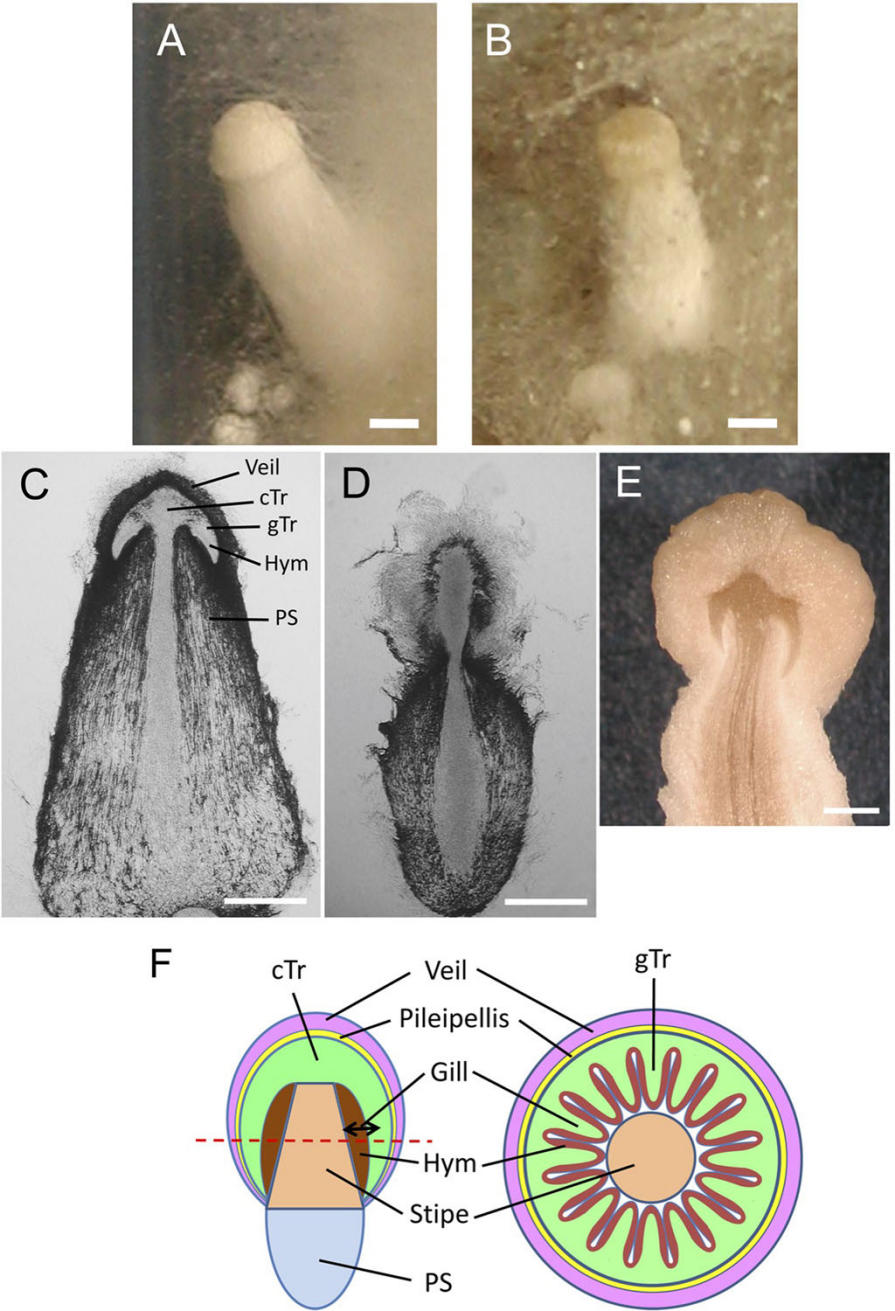
**The *cag1-1* mutant phenotypes.** (A) Wild-type fruiting body primordium. (B) *cag1-1* mutant primordium. (C) Vertical section of the wild-type primordium. The rudimentary pileus differentiates on the primordial shaft (PS). Veil cells cover the upper region of the pileus. From the central trama tissue (cTr), the gill trama tissue (gTr) protrudes to produce gills at the underside of the pileus. The surface of the gills comprises the hymemium (Hym). (D) Vertical section of the *cag1-1* mutant primordium. (E) The mutant primordium occasionally enlarges without gills. Scale bars: 1 mm. (F) Schematic diagrams of tissues in the fruiting body primordium at the stage after 10–36 h from the time when the light trigger for maturation is received.

As the fruiting body primordia enlarge, the gills protrude from the central trama tissue of the pileus toward the primordial shaft (Fig. 1). Some cells at the surface of the gills differentiate into basidia, where meiosis occurs and on which basidiospores are formed (Burns et al., 2010), whereas the others differentiate into paraphyses (Rosin and Moore, 1985). How the agaric gills develop has been observed in both *C. cinerea* and *Volvariella bombycina* (Chiu and Moore, 1990), but little is known about the molecular mechanisms underlying gill formation and the differentiation of basidia.

*Saccharomyces cerevisiae* Tup1p has been characterised as a transcriptional corepressor, which forms a complex with Cyc8 (Ssn6) to exert its function (Tzamarias and Struhl, 1994). Tup1 homologues are conserved throughout eukaryotes (Courey and Jia, 2001) and have been demonstrated to regulate gene expression in a wide variety of cellular processes, including metabolic change, asexual and sexual development, responses to environmental signals and developmental switching (Elías-Villalobos et al., 2011; Hicks et al., 2001; Long et al., 2006; Todd et al., 2003; Yamashiro et al., 1996).

To understand the molecular mechanisms that underlie the development of the pileus in *C. cinerea*, we previously investigated the *cap-growthless* mutant, #299, which carries a recessive mutation, *cap-growthless1-1* (*cag1-1*) (Kemuriyama and Muraguchi, 2014). The *cag1-1* mutant fails to develop the gills and terminates fruiting, thereby producing the primordia that never mature and that lack basidia in the cap-like structure. In the present study, we found that the *cag1* gene encodes a Tup1 homologue. We also examined the expression levels, subcellular localisation and tissue distribution of Cag1 and its paralogue Cc.TupA in fruiting body development.

## RESULTS

### *cag1-1* mutant phenotypes

We mutagenised a homokaryotic fruiting strain of *C. cinerea*, #326, by UV-irradiation and screened for developmental mutants. The mutant strain, #299, exhibited delayed primordium formation and the maturationless phenotype, where the small fruiting body primordia formed but never entered the maturation stage (Fig. 1B). The upper region of the primordia bulged to form a pileus-like structure in appearance, but the gill structure was not discernible in vertical sections of the mutant primordia (Fig. 1D,E). The mutant phenotype was designated as *cap-growthless*. Genetic analysis of #299 indicated that the mutant phenotype was caused by a single recessive mutation, *cap-growthless1-1* (*cag1-1*) (Kemuriyama and Muraguchi, 2014). In continuous dark conditions, the *cag1-1* mutant strain could exhibit the so-called dark stipe phenotype, which the wild-type strain exhibits in continuous dark conditions (data not shown), thereby suggesting that the light reception system is formed in this mutant.

### Identification of the *cag1* gene

**Fig. 2.**
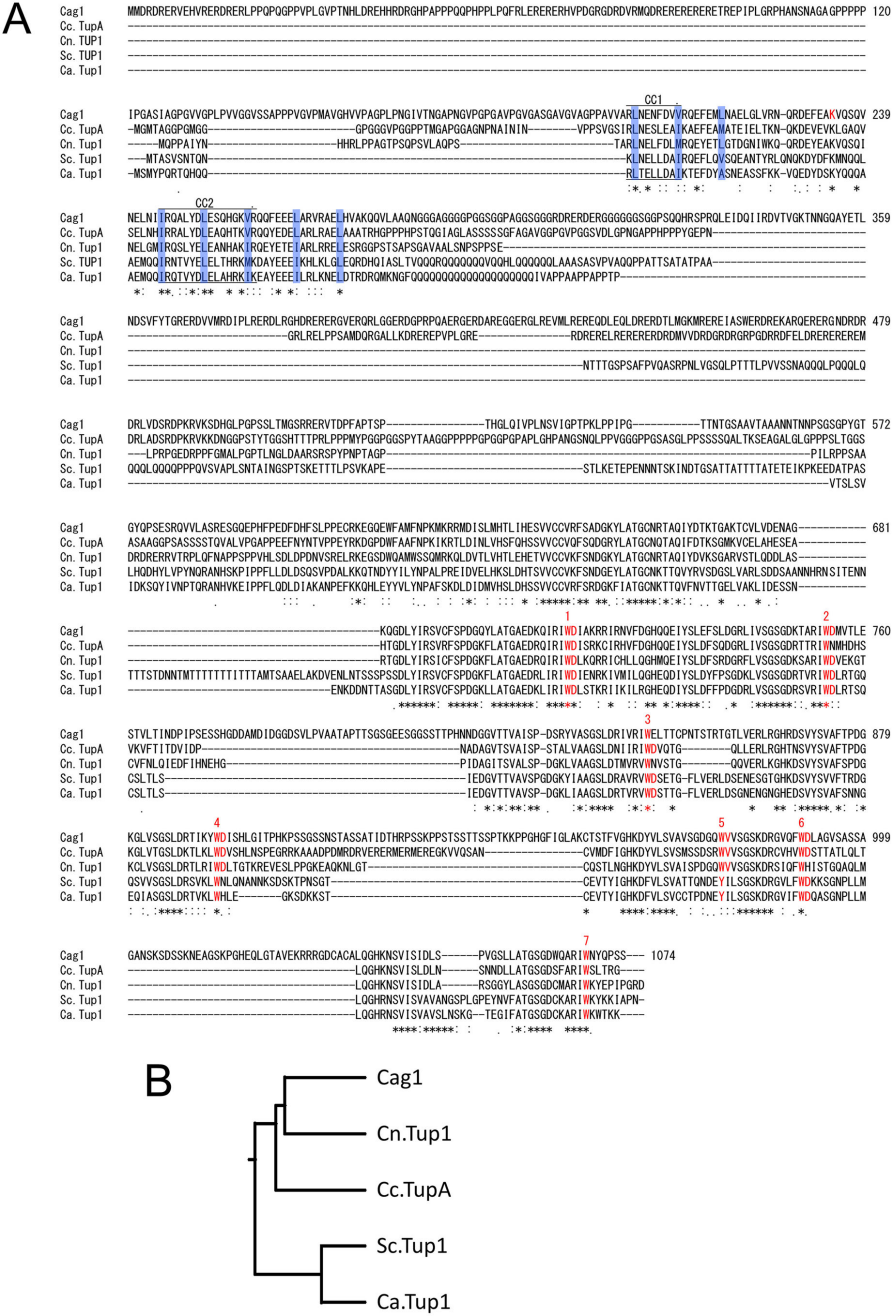
**Cag1 and Cc.TupA structures.** (A) Alignment of the amino acid sequences of the Cag1 and Tup1 orthologues in other fungi. Amino acid sequences of Tup1 orthologues were obtained from NCBI and the Broad Institute databases and aligned by CLUSTALW. Cc, *Coprinopsis cinerea*; Cn, *Cryptococcus neoformans*; Um, *Ustilago maydis*; Ag, *Ashbya gossypii*; An, *Aspergillus nidulans*; Sc, *Saccharomyces cerevisiae*. Gene numbers of Cn and Um are based on the Broad Institute database. Two coiled-coil regions, CC1 and CC2 are indicated in the Tup_N domain. Lysine 234 indicated by the red letter was mutated to a stop codon in *cag1-1*. WD40 repeats are also indicated by the red letters with the repeat number. (B) Phylogenetic tree of Cag1 and Tup1 homologues in fungi.

The *cag1* locus was mapped to chromosome IX using random amplified polymorphic DNA (RAPD) markers (Kemuriyama and Muraguchi, 2014). To identify the *cag1* gene, we transformed the *cag1-1* mutant strain #58 with BAC DNA carrying genomic fragments of chromosome IX (Stajich et al., 2010) and found that BAC DNA s7H8 complemented the *cag1-1* mutation (Kemuriyama and Muraguchi, 2014). A subclone derived from s7H8, B4, could also rescue the mutation, thereby narrowing the complementing region to ca 50 kb. The rescue activity was retained after *Hin*dIII digestion of B4 DNA, so relatively large *Hin*dIII fragments were subcloned and examined to assess their rescue activity. An 8 kb *Hin*dIII fragment possessed the rescue activity, and two genes were predicted within the fragment. When the 8 kb *Hin*dIII fragment was digested with *Apa*I, which cleaves one gene, the rescue activity was not lost. The other gene, which lacked an *Apa*I site, was CC1G_08590 in the *Coprinopsis cinerea* database of the Broad Institute, so we hypothesised that this gene was *cag1*. To confirm this hypothesis, we searched for a mutation site in the mutant gene and found a single A-to-T nonsense mutation in the *cag1-1* mutant (Fig. S1). This mutation changed a lysine at codon 234 into a stop codon in the predicted protein (Fig. 2), thereby suggesting loss of function of the N-terminal domain.

### The *cag1* gene encodes a Tup1 homologue

The *cag1* gene (CC1G_08590) encodes a Tup1 homologue. A Blast Coprinus Genome database (available at: http://genome.semo.edu/cgi-bin/blastall_new.pl) search demonstrated that the *C. cinerea* genome contains another Tup1 homologue gene (CC1G_08510), which we designated as *Cc.tupA*. These Tup1 paralogues are present on chromosome IX. Fig. 2A shows an alignment of the Tup1 homologues. Cag1 has the longest N-terminal region compared with other Tup1 homologues. The N-terminal region of these Tup1 homologues possesses the conserved domain, Tup_N, which contains two coiled-coil regions (CC1 and CC2 in Fig. 2A). The *cag1-1* mutation occurred between CC1 and CC2. The presence of the Tup_N domain in Cag1 suggests that Cag1 self-assembles to form a tetramer as Tup1p in *S. cerevisiae* (Jabet et al., 2000; Matsumura et al., 2012; Varanasi et al., 1996). Cag1 contains seven WD40 repeats at the C-terminal region like other Tup1 homologues.

### Expression of *cag1* increases in the pileus

**Fig. 3.**
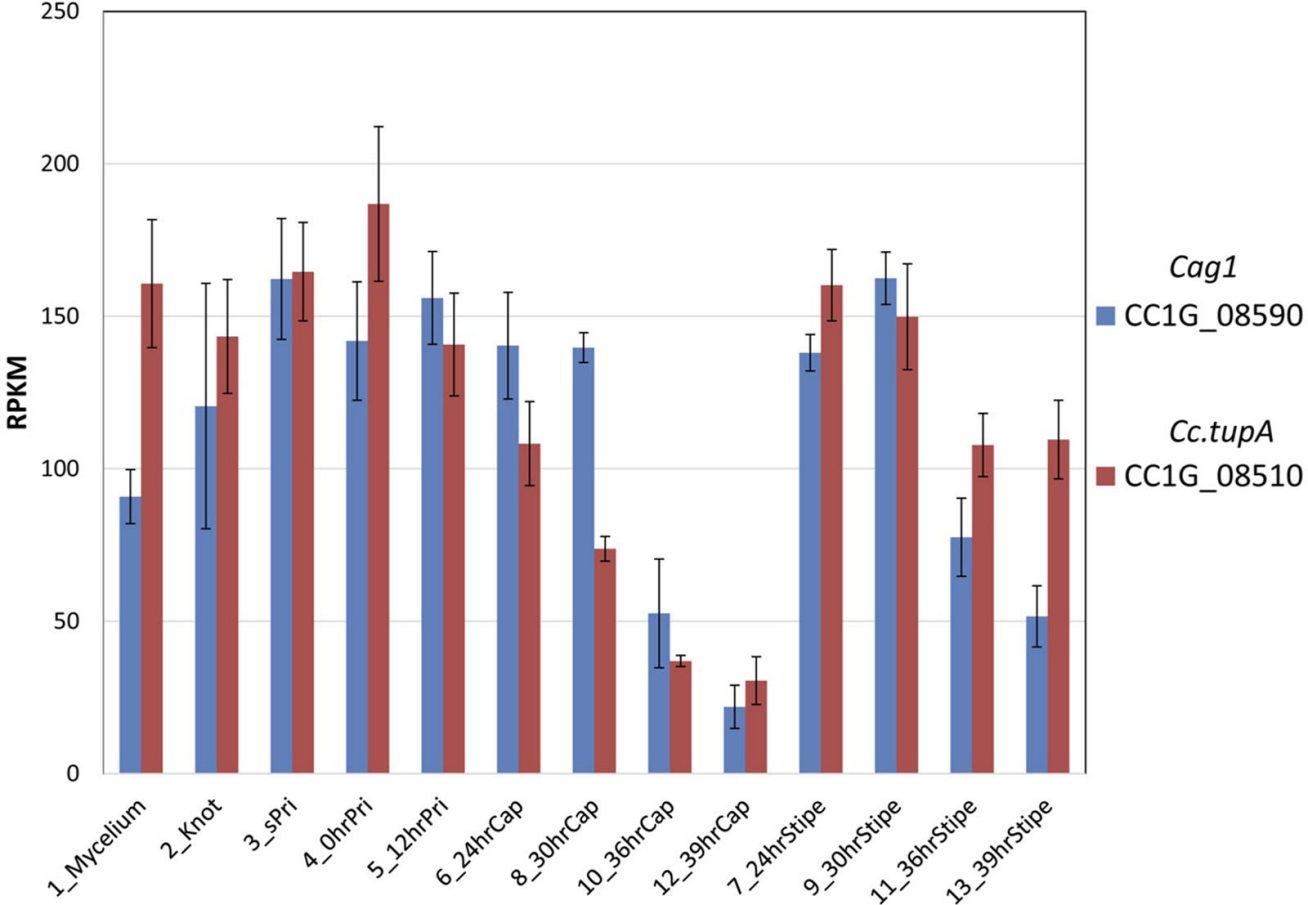
**Expression analysis of *cag1* and *Cc.tupA*.** Expression levels of *cag1* and *Cc.tupA* according to RNA-seq analysis (Muraguchi et al., 2015). Expression levels are shown as reads per kilobase per million (RPKM) values.

We performed RNA-seq analyses of fruiting body development using samples from 13 stages/tissues (Muraguchi et al., 2015). Based on the RNA-seq data, we examined whether the expression levels of *cag1* and *Cc.tupA* are developmentally regulated during fruiting. In the vegetative mycelium, *Cc.tupA* was expressed more than *cag1*. In contrast, in the pileus at the 12–36-h stage, the *cag1* expression levels were higher than those of *Cc.tupA* (Fig. 3). The increase in *cag* expression levels in the pileus was confirmed by quantitative real-time PCR (Fig. S2). This change in the *cag1* expression levels suggests that the function of Cag1 is required for pileus growth, which is consistent with the finding that the *cag1* mutation causes the cap-growthless phenotype.

### Cag1 is preferentially localised in the nucleus of the gill trama tissue cells

To examine changes in the spatial and temporal expression of Cag1 and Cc.TupA in the pileus, we reciprocally fused fluorescent tags, i.e. EGFP and mCherry, to these proteins. Reciprocal tagging can circumvent two problems in this fungus: the random integration of introduced DNA fragments into the *C. cinerea* genome and autofluorescence in the pileus (Fig. S3). Reciprocal tagged proteins should be expressed from random integration sites in the *C. cinerea* genome, so if they exhibit similar localisation, then this should be attributable to the nature of the genes and proteins. Some types of cells in the pileus exhibit blue, green and red autofluorescence, which should be common even in cells expressing reciprocally tagged proteins.

**Fig. 4.**
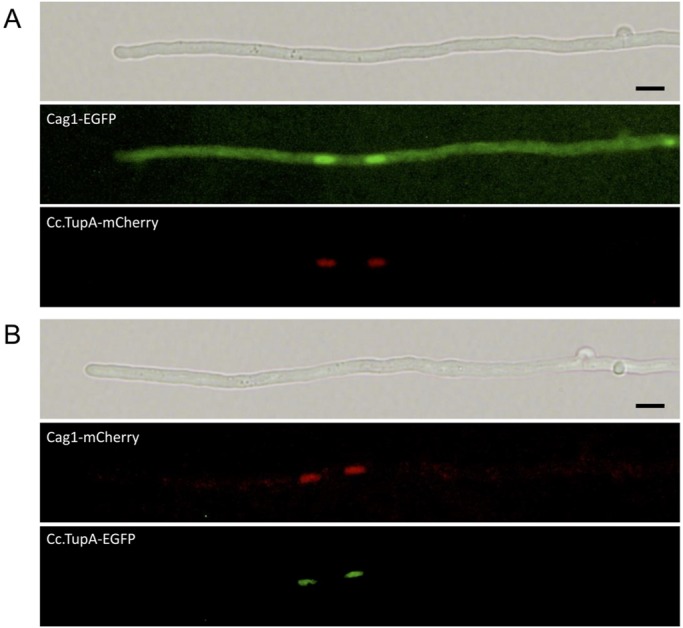
**Cag1 and Cc.TupA in vegetative hyphae.** (A) Dikaryotic vegetative hypha expressing Cag1-EGFP and Cc.TupA-mCherry. (B) Dikaryotic vegetative hypha expressing Cag1-mCherry and Cc.TupA-EGFP. Scale bars: 5 µm.

The tagged Cag1 proteins, Cag1-EGFP and Cag1-mCherry, were expressed in the *cag1-1* mutant strain, and they rescued the mutation, thereby confirming that the tagged Cag1 proteins were functional. The tagged Cag1 and Cc.TupA were observed in the nuclei of vegetative mycelia (Fig. 4), which also suggested that the tagged Cc.TupA was functional. The nuclear localisation of Cag1 and Cc.TupA in the vegetative mycelium suggests that Cag1 and Cc.TupA function in the nucleus, which is supported by the finding that Tup1p of *S. cerevisiae* functions as a corepressor or coactivator to regulate gene expression.

Because RNA-seq data indicated that the *cag1* expression levels were increased in the pileus (Fig. 3), we observed the tagged Cag1 and Cc.TupA in the developing pileus tissue. The pileus of the *C. cinerea* fruiting body comprises at least three tissues (Fig. 1C,F): (1) veil cells covering the upper surface of the pileus; (2) trama tissue, which occupies the central inner part of the pileus and continues to the medial part of gills and (3) the hymenium, which is a layer of cells that includes basidia and covers the surface of gills. In basidia, nuclear fusion occurs at the 12–24-h stage, followed by meiosis in the 24–36-h stage and sporulation in the 36–39-h stage. In the sporulation stage, paraphyses differentiate among basidia (Rosin and Moore, 1985).

**Fig. 5.**
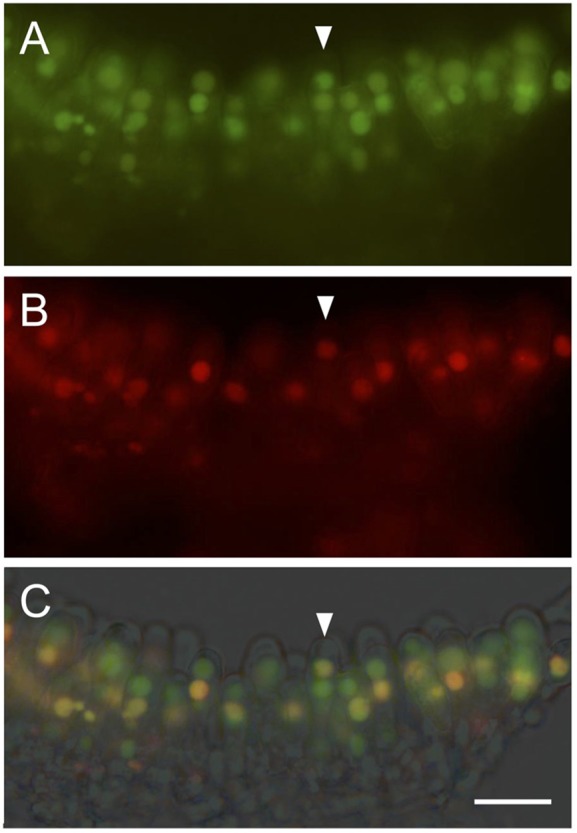
**Cc.TupA-EGFP and green autofluorescence in the basidia**. (A) As the basidia matured, green autofluorescent compounds were accumulated by vacuoles in the basidia. EGFP signals from Cc.TupA-EGFP were observed in the nuclei of the basidia. In the nucleus, the dark spot corresponds to the nucleolus. (B) The nuclei were labelled with mCherry-Cc.Sumo1. Arrowheads indicate a basidium with the nucleus above the vacuole. Scale bar: 20 µm.

As the fruiting body matured, vacuoles developed in the basidia and started to emit green autofluorescence (Fig. S3). The green autofluorescence made it difficult to distinguish between the nucleus with EGFP signals and the vacuoles that developed in a basidium. However, the EGFP signals could be distinguished from the autofluorescence because the autofluorescence had a longer wave length than the EGFP signals (Fig. 5A). The nucleus with EGFP signals had a dark spot, which was the nucleolus, whereas this dark spot was not discernible in vacuoles. To verify the localisation of Cc.TupA-EGFP in the nuclei of basidia, we used mCherry-tagged Cc.Sumo1 (CC1G_04810, 100 amino-acid protein) to visualise the nucleus. It is known that Sumo conjugation mostly occurs with nuclear proteins (Wong et al., 2008). Fig. 5B shows basidia that expressed mCherry-Cc.Sumo1 and Cc.TupA-EGFP at the 30-h stage when the nucleus undergoes meiosis I. Most of the basidia had vacuoles at the tip region, but the position was occasionally reversed (arrowheads in Fig. 5). Thus, the nuclear localisation of Cc.TupA-EGFP was evident in the basidia.

**Fig. 6.**
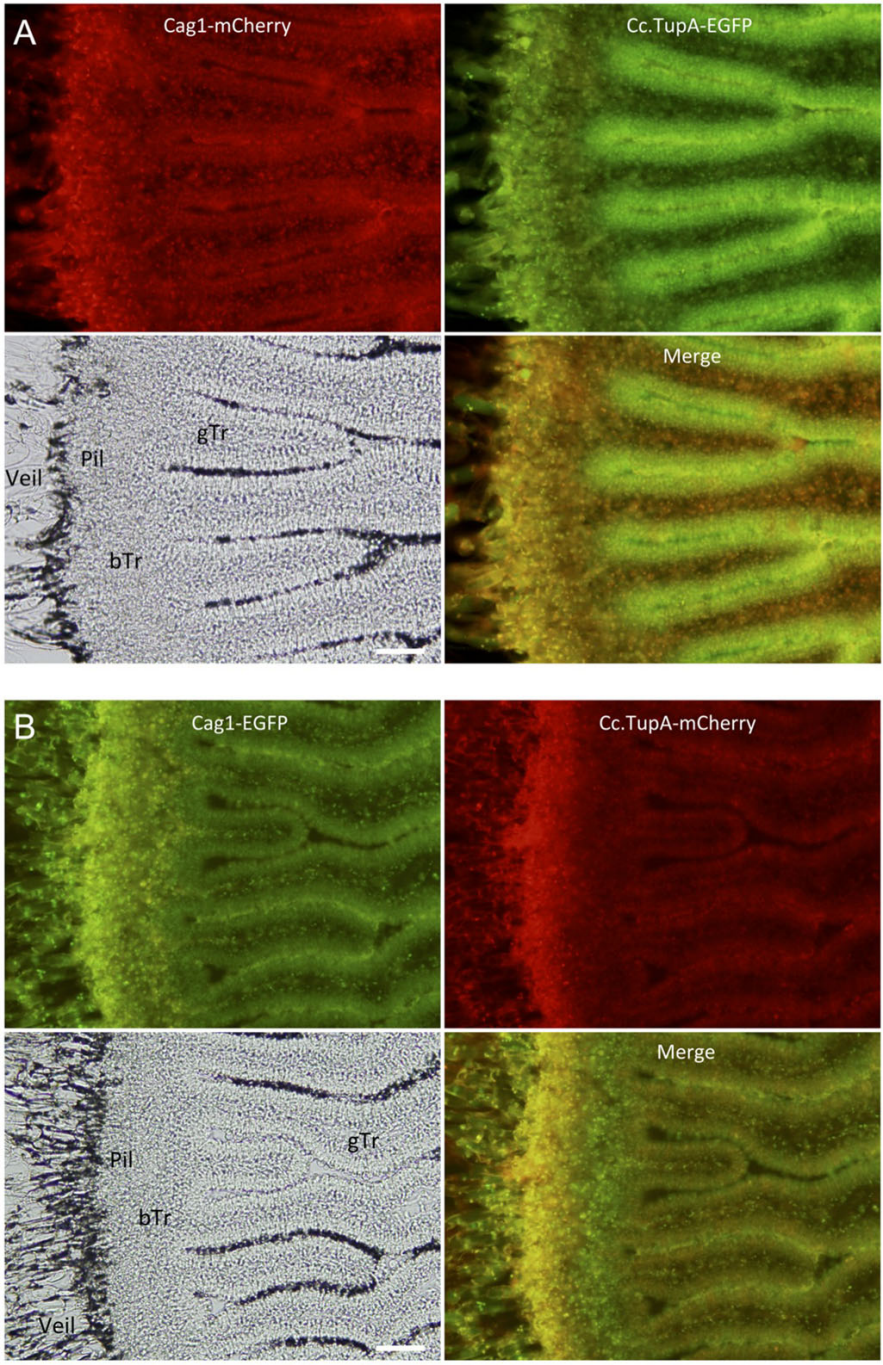
**Cag1 and Cc.TupA in the pileus at the 10-h stage.** Transverse section of the 10-h stage pileus. (A) Cag1-mCherry and Cc.TupA-EGFP were expressed. (B) Cag1-EGFP and Cc.TupA-mCherry were expressed. The veil, pileipellis (Pil), basal trama (bTr) and gill trama (gTr) are indicated in the bright field panel. Scale bars: 50 µm.

**Fig. 7.**
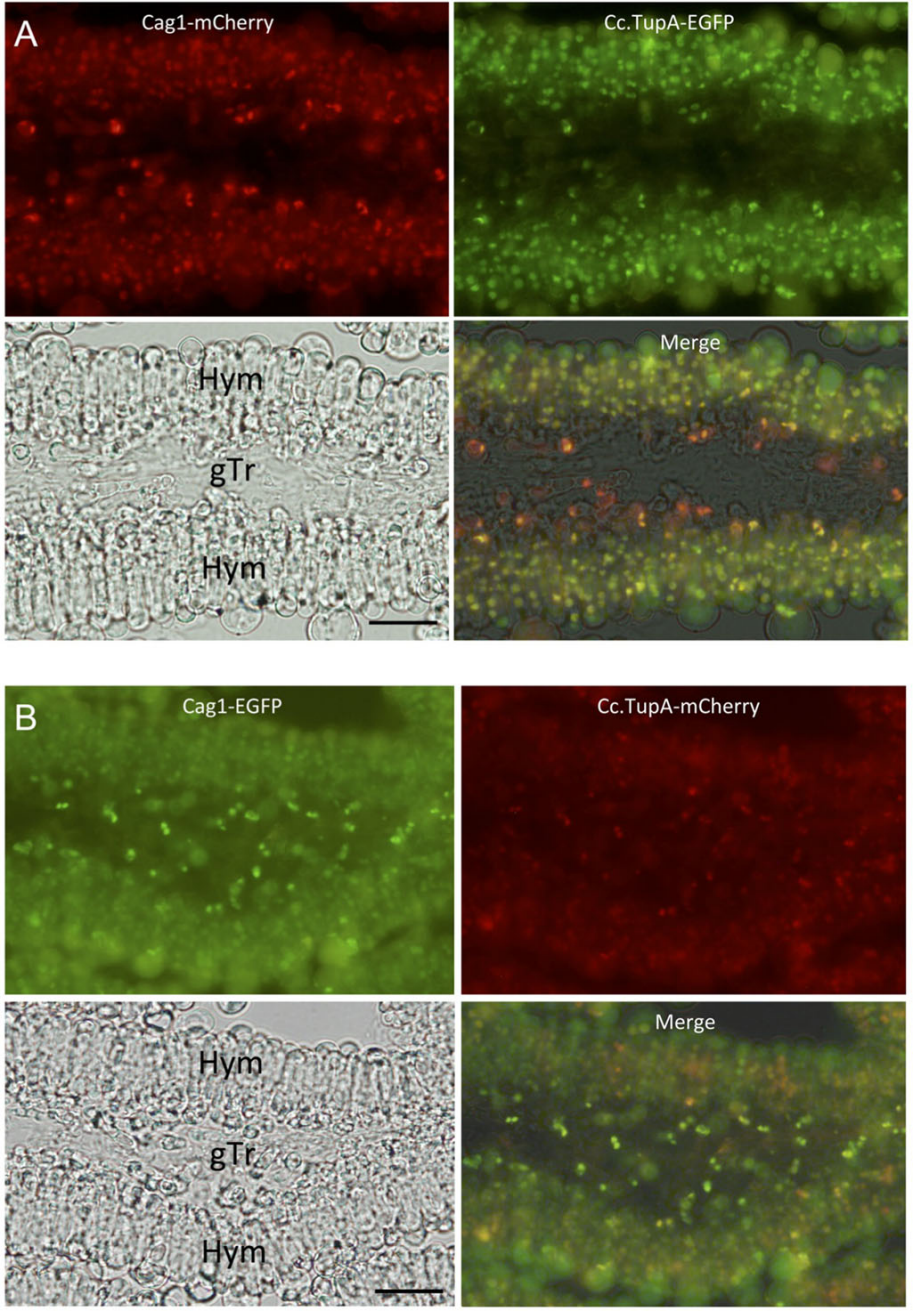
**Cag1 and Cc.TupA in the gill at the 10-h stage.** Transverse section of the 10-h stage gill. Two nuclei that will fuse in karyogamy are apparent in a basidium. (A) Cag1-mCherry and Cc.TupA-EGFP were expressed. (B) Cag1-EGFP and Cc.TupA-mCherry were expressed. The gill trama (gTr) and hymenium (Hym) are indicated in the bright field panel. Scale bars: 20 µm.

**Fig. 8.**
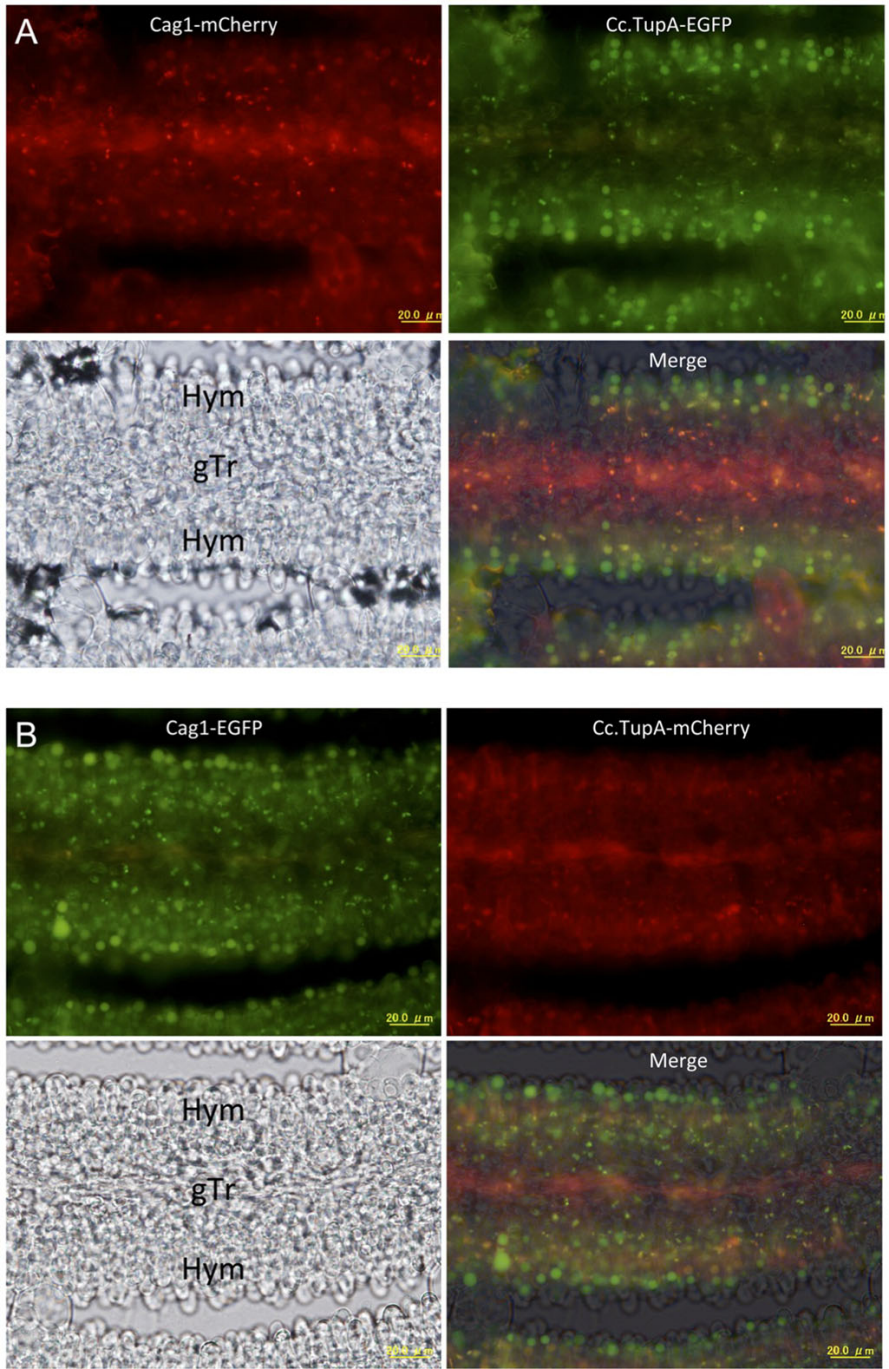
**Cag1 and Cc.TupA in the gill at the 30-h stage.** Transverse section of the 30-h stage gill. A single nucleus is visible in a basidium. (A) Cag1-mCherry and Cc.TupA-EGFP were expressed. (B) Cag1-EGFP and Cc.TupA-mCherry were expressed. The gill trama (gTr) and hymenium (Hym) are indicated in the bright field panel. Scale bar: 20 µm.

In sections of the pileus, we could observe cells that expressed both the tagged Cag1 and Cc.TupA. From the small primordial stage until the 30-h stage, the fluorescence of mCherry and EGFP indicated that Cag1 and Cc.TupA were co-localised in the nuclei of cells in the pileipellis, which is the cortical layer of the pileus (Fig. 6). Co-localisation was also observed in the veil cells. At the 10-h stage when two nuclei will fuse during karyogamy in a basidial cell, co-localisation was also observed in the basidia and sub-hymenium cells (Fig. 7). At the 30-h stage, the co-localisation was also observed in the nuclei of the basidia (Fig. 8). Compared with Cc.TupA, Cag1 was preferentially expressed in the nuclei of the gill trama tissue cells (Figs 6, 7 and 8). This Cag1 expression pattern was consistent with the mutant phenotype due to the lack of gills in the *cag1-1* mutant.

**Fig. 9.**
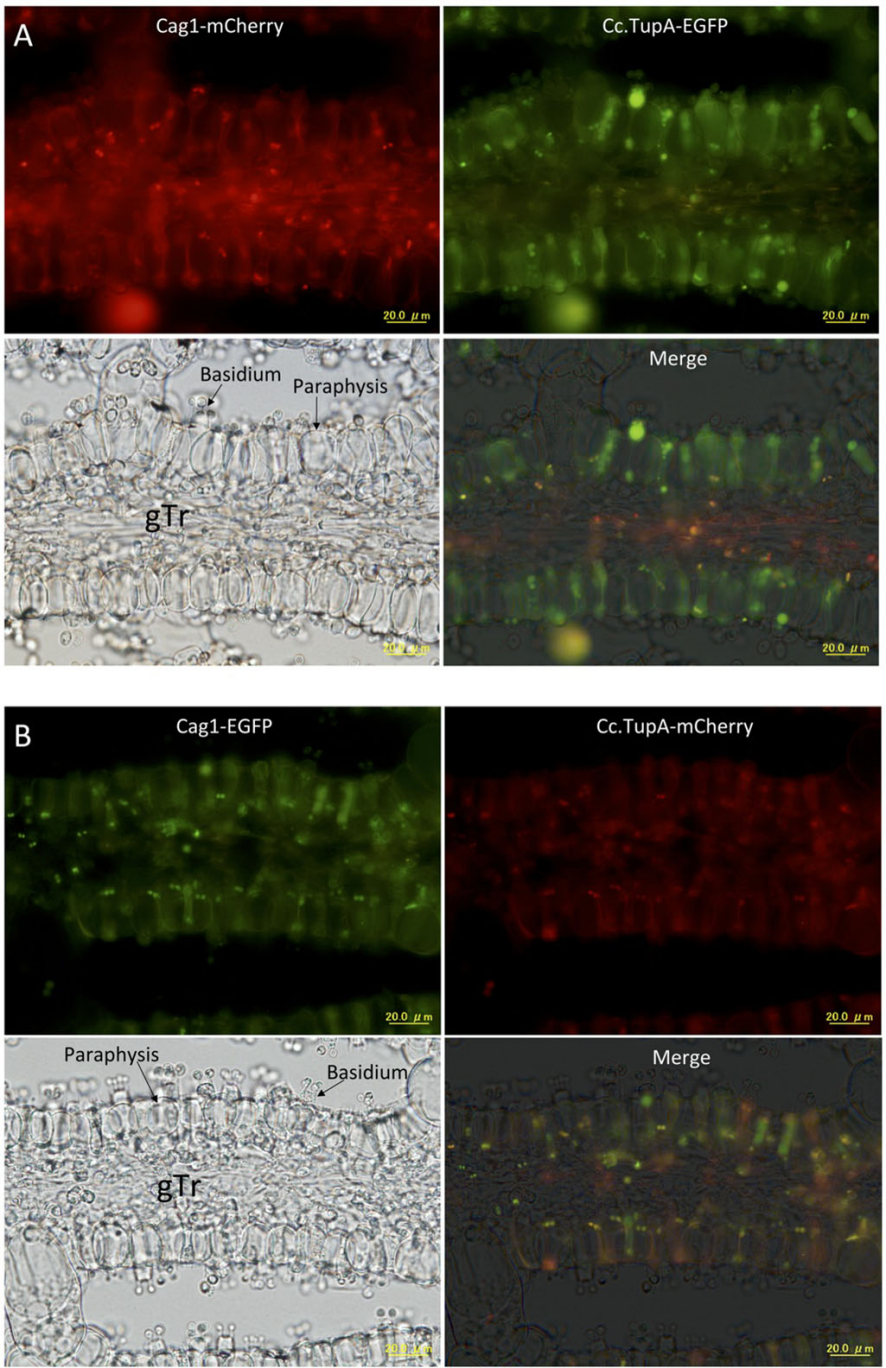
**Cag1 and Cc.TupA in the gill at the 39-h stage.** Transverse section of the 39-h stage gill. Four basidiospores are produced on a basidum, and paraphysis cells are visible among basidia. (A) Cag1-mCherry and Cc.TupA-EGFP were expressed. (B) Cag1-EGFP and Cc.TupA-mCherry were expressed. The basidium and paraphysis are indicated in the bright field panel. Scale bars: 20 µm.

During the sporogenesis stage, paraphysis cells become discernible in the hymenium (Fig. 9). In the sub-hymenial region, young paraphyses arise as branches from the sub-basidial cells during meiotic nuclear division and are inserted among the basidia (Rosin and Moore, 1985). We found that two paired nuclei were positioned at the basal part of the paraphysis cells, and they contained both Cag1 and Cc.TupA (Fig. 9).

### Cag1 might function by interaction with Cc.TupA

**Fig. 10.**
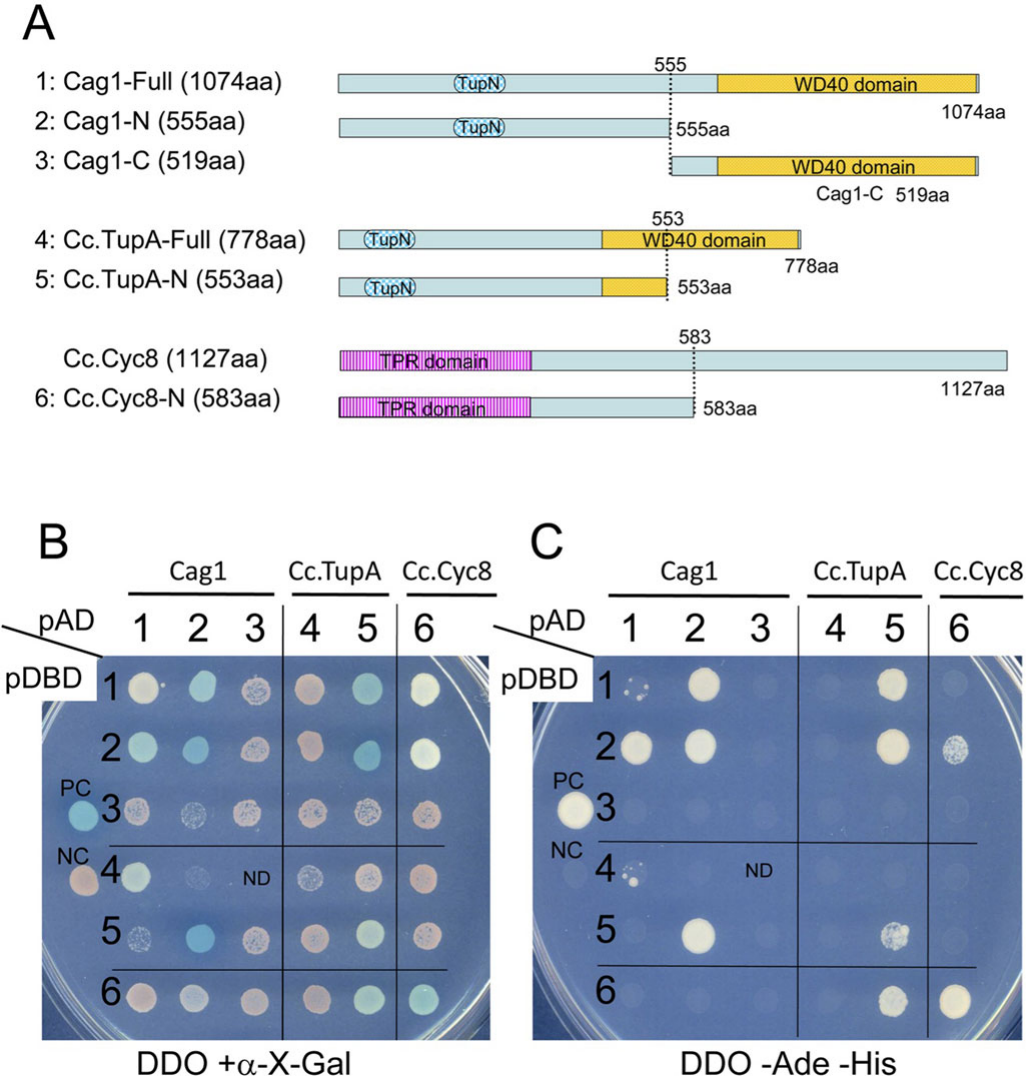
**Yeast two-hybrid assays of Cag1, Cc.TupA and Cc.Cyc8.** (A) Strain Y2HGold was transformed with constructs encoding fusion proteins of the DNA-binding domain (BD) of *GAL4* with the six proteins indicated. Strain Y187 was also transformed with constructs encoding fusion proteins of the activation domain (AD) of *GAL4* with the six proteins indicated. (B,C) Transformed strains were mated with each other, and the resulting diploids were selected on SD/-Trp/-Leu (DDO) medium before spotting onto DDO/+α-X-Gal and DDO/-Ade/-His to assess activation of the *MEL1* reporter (B) or the *ADE2* and *HIS3* reporters (C). ND indicates no growth of mated cells.

Reciprocal tagging of Cag1 and Cc.TupA indicated the co-localisation of these two Tup1 paralogues in the nuclei of vegetative mycelium, pileipellis, veil cells, and paraphyses. These co-localisation results, as well as the fact that Tup1p of *S. cerevisiae* interacts with itself via the Tup_N domain (Jabet et al., 2000; Matsumura et al., 2012; Varanasi et al., 1996) and that Cag1 and Cc.Tup1 both have the Tup_N domain, prompted us to examine whether Cag1 interacts with Cc.TupA. In *S. cerevisiae*, the N-terminal domain of Tup1p also interacts with Cyc8p (Jabet et al., 2000; Varanasi et al., 1996). Using a yeast two-hybrid (Y2H) assay, we examined the interactions among Cag1, Cc.TupA and the N-terminal region of Cc.Cyc8 using six fusion proteins (Fig. 10A). The N-terminal region of Cag1 (2 in Fig. 10) and the N-terminal region of Cc.TupA (5 in Fig. 10) could self-assemble independently of the fused domains. In addition, the N-terminal region of Cag1 (2 in Fig. 10) strongly interacted with the N-terminal region of Cc.TupA (5 in Fig. 10) independently of the fused domains. The strong interaction according to the Y2H assay results suggests that Cag1 and Cc.TupA may interact with each other to exert their functions in the specific cells that exhibited co-localisation. As expected, the N-terminal region of Cc.Cyc8 (6 in Fig. 10) could interact with the N-terminal region of Cag1 (2 in Fig. 10) or Cc.TupA (5 in Fig. 10), although the interactions were affected by the fused domains. These results suggest that Cc.Cyc8 interacts with Cag1 or Cc.TupA to form a complex in *C. cinerea* cells, as found in *S. cerevisiae*.

## DISCUSSION

We have investigated a *cap-growthless1-1* (*cag1-1*) mutant, which produces fruiting body primordia that never mature. The mutant primordia failed to produce gills in the pileus-like structure. In this study, we showed that the *cag1* gene (CC1G_08590) encodes a Tup1 homologous protein. The *cag1-1* mutant has a nonsense mutation in the Tup-N domain, thereby suggesting that this mutation causes the complete loss of Cag1 function. If Cag1 functions as a transcriptional corepressor, then loss of the Cag1 function should cause the expression of certain genes. However, the *cag1-1* mutant lacks gills in the pileus, which suggests that Cag1 activates the expression of genes required for gill formation.

Tup1 homologues have also been studied in filamentous fungi and are implicated in switching of cell growth, conidiation and pathogenesis in *Neurospora crassa* (Yamashiro et al., 1996), *Candida albicans* (Braun and Johnson, 1997, 2000), *Aspergillus nidulans* (Hicks et al., 2001), *Penicillium marneffei* (Todd et al., 2003), *Cryptococcus neoformans* (Lee et al., 2005, 2009) and *Ustilago maydis* (Elías-Villalobos et al., 2011). These studies on some phenotypes suggested that Tup1 homologues activate the expression of certain genes. A recent study on *S. cerevisiae* Tup1p suggested that Tup1p itself functions as both a corepressor and coactivator (Chen et al., 2013); therefore, it is possible that Cag1 functions as a coactivator of gill formation.

The *C. cinerea* genome harbours a paralogue of *cag1*, *Cc.tupA* (CC1G_08510). The mutant phenotypes indicated that Cag1 and Cc.TupA play different roles. Reciprocal tagging analyses demonstrated the distributions of cells expressing Cag1 and Cc.TupA, where the co-localisation of these paralogues was observed in the nuclei of cells in the vegetative hyphae, pileipellis, basidia and paraphyses. The results of Y2H analyses showed that the N-terminal regions of Cag1 and Cc.TupA interact strongly with each other, so it is possible that Cag1 interacts with Cc.TupA in the nucleus where they are co-localised. However, the loss of Cag1 function did not affect vegetative hyphal growth and the early stage of fruiting, i.e. hyphal knot formation, as well as the differentiation of the veil and primordial shaft cells. This suggests that Cag function is dispensable for these cells, at least in normal conditions.

Compared with Cc.TupA, Cag1 was preferentially expressed in cells of the gill trama tissue cells, which was consistent with the lack of gills in the *cag1-1* mutant primordia. A high ratio of Cag1 to Cc.TupA might promote the expression of genes required for differentiation and the growth of gill trama tissue. Future research will be required to identify the genes where their expression is activated by Cag1. Cc.TupA was expressed in the basidia, which are considered to be hyphal tips. Thus, the function of Cc.TupA might be required to restart tip growth to develop basidia from the gill trama. Further research will also be required to identify the genes with expression levels that are influenced by Cc.TupA. The yeast Tup1-Cyc8 protein complex has been demonstrated to interact with various molecules, including transcription factors (Komachi et al., 1994), under-acetylated histones H3 and H4 (Edmondson et al., 1996), class I and II histone deacetylases (Watson et al., 2000; Wu et al., 2001) and phosphoinositide lipid PI(3,5)P_2_ (Han and Emr, 2011). These interacting molecules require further investigation to understand the molecular mechanisms that facilitate gill trama and basidia development.

Previously, we identified the *ich1* gene and showed that its mutation blocks pileus formation (Muraguchi and Kamada, 1998). The morphology of *ich1-1* mutant primordia differs from that of the *cag1-1* mutant. The differences in morphology between these two mutants suggests that the function of Ich1 is required in an earlier stage than that of Cag1. Ich1 might allow the pileipellis region to expand, whereas Cag1 might be required for protrusion of the gill trama tissue from the expanded pileipellis toward the stipe. Thus, to understand the molecular mechanisms that underlie pileus formation in mushrooms, it will also be important to examine the molecular relationship between the functions of Ich1 and Cag1.

## MATERIALS AND METHODS

### Strains, culture conditions and genetic techniques

The *C. cinerea* strains used in this study are listed in Table 1. Strain #299 is the *cag1-1* recessive mutant, which was induced by UV mutagenesis of a homokaryotic fruiting strain #326. The dikaryotic strain 5026+5132 was used as the wild type for fruiting. Malt extract–yeast extract–glucose (MYG) medium (Rao and Niederpruem, 1969) solidified with 1.5% (w/v) agar was used in all experiments. MYG slant medium in test tubes was used to observe the fruiting phenotypes. Basidiospore germlings were isolated at random using a chisel-shaped needle under a dissecting microscope (Miles et al., 1966).

**Table 1.**
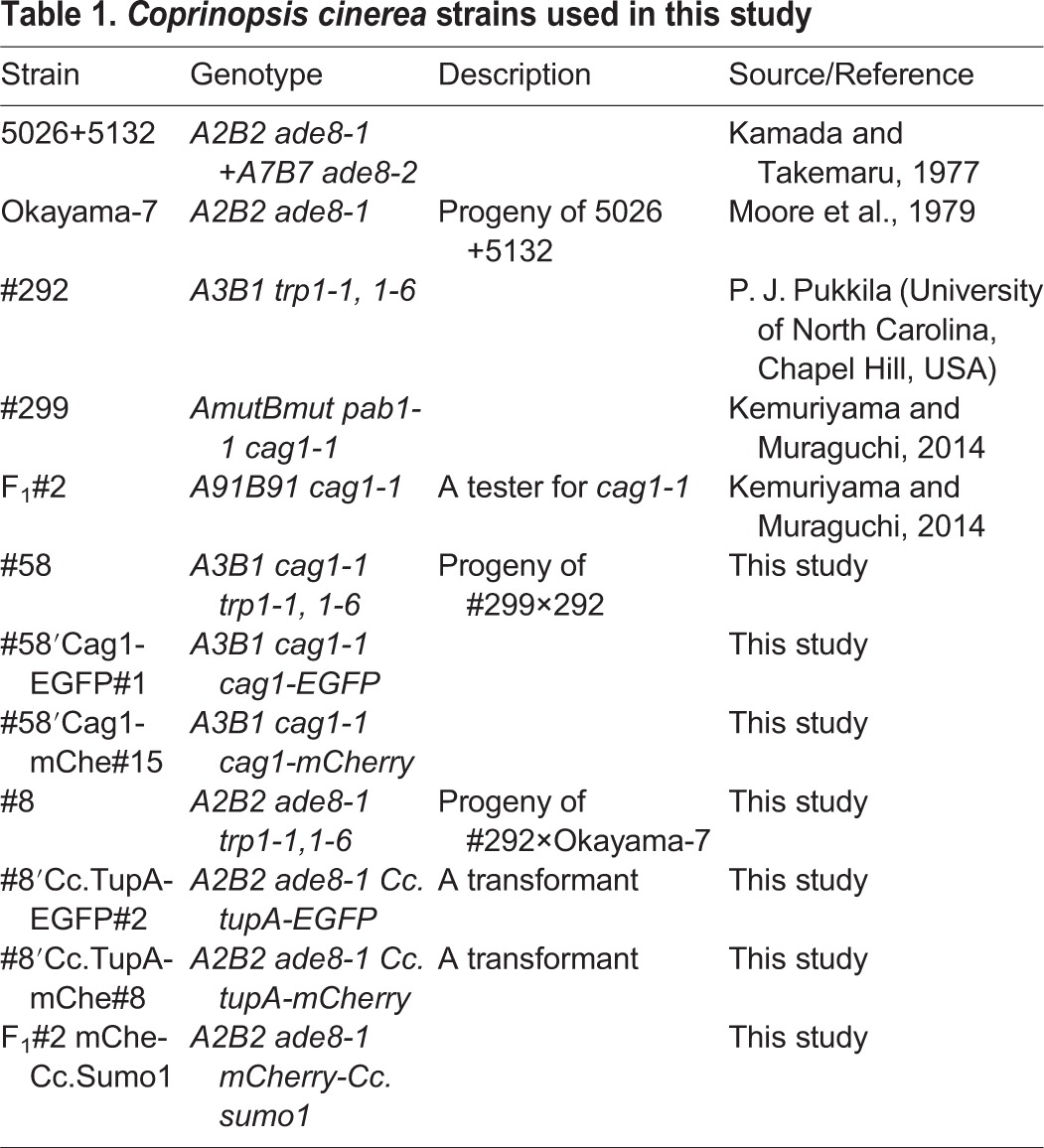
***Coprinopsis cinerea* strains used in this study**

### Transformation experiments

To obtain a recipient strain for the transformation experiments, the original mutant strain #299 (*AmutBmut pab1-1 cag1-1*) was crossed with #292 (*A3B1 trp1-1,1-6*). Among the F_1_ progeny, strain #58 (*A3Bm trp1-1,1-6 cag1-1*) was selected as the recipient strain. Tester strain F_1_#2 (*A91B91 cag1-1*) was selected from among the F_1_ progeny derived from a cross between #299 and KF_3_#2 (*A91B91*). Protoplasts of strain #58 were obtained from oidia and transformed with BACs as described previously (Binninger et al., 1987; Muraguchi et al., 2005). Trp^+^ transformants were crossed with tester strain F_1_#2 in MYG slant medium to observe the fruiting phenotype.

The BAC DNA of s7H8 was digested partially with *Hin*dIII and fractionated using CHEF electrophoresis. The gel portion containing fragments >40 kb was excised and subjected to electroelution as described previously (Muraguchi et al., 2005). The recovered fragments were self-ligated and transformed into competent DH10B cells to construct a sub-library. The DNA was extracted from the subclones of s7H8 and examined to assess their complementing activity. Subclone B4 exhibited the complementing activity, thereby narrowing the region with the activity to within about 50 kb.

### Quantitative real-time PCR (qRT-PCR)

Total RNA was extracted from the vegetative mycelium, fruiting body primordia, stipe and pileus using RNAiso solution (Takara Co.). The fruiting primordia were harvested between the 0-h and 12-h stage (Muraguchi et al., 2015). The pileus and stipe tissues were harvested from the fruiting bodies around the 36-h stage. cDNAs for qRT-PCR were synthesised from the total RNAs using a RevaTra Ace qPCR RT Kit (TOYOBO). Gene expression was quantified with a CFX96 system (Bio-Rad). The primers used for qRT-PCR are listed in Table S1.

### DNA sequencing

Genomic DNA was extracted as described previously (Zolan and Pukkila, 1986). The coding region of the *cag1* gene was amplified using four sets of primers (Table S1) with iProof DNA polymerase (Bio-Rad), subjected to agarose gel electrophoresis, purified from the agarose gels with GENECLAEN II Kit (Bio101), and used as templates for cycle sequencing reactions with BigDye Terminator v3.1 (Applied Biosystems). Sequencing was performed by the Biotechnology Center at Akita Prefectural University.

### Construction of strains expressing fluorescent protein-tagged Cag1, Cc.TupA and Cc.Sumo1

All of the DNA manipulations were performed according to standard methods (Sambrook and Russell, 2001) with DH10B or HTS08 (Takara Bio) as the host. The oligonucleotide primers used are listed in Table S1. To construct p*cag1-*mCherry, the fragment containing the *cag1* native promoter and the coding region was amplified by iProof DNA polymerase (Bio-Rad) using primers *Hin*dIII-cag1(P)-For and *Bam*HI-cag1-Rev and inserted into the pmCherry plasmid (Clontech) digested by *Hin*dIII and *Bam*HI. The fragment of the *cag1* terminator was amplified using primers *Not*I-*cag1*(T)-For and *Not*I-*cag1*(T)-Rev and inserted into the *Not*I site in the pmCherry plasmid carrying the *Hin*dIII and *Bam*HI fragment, thereby yielding p*cag1*-mCherry. To replace the mCherry region with EGFP, the same strategy was used for the pEGFP-1 plasmid (Clontech), which yielded p*cag1*-EGFP.

To tag Cc.TupA with EGFP, genomic DNA containing the *Cc.tupA* promoter and the coding region was amplified to carry restriction sites at both ends and inserted into the *Hin*dIII and *Kpn*I sites of pEGFP-1. The *Cc.tupA* terminator was also amplified to carry restriction sites at both ends and inserted into the *Not*I and *Xba*I sites of pEGFP-1. To replace EGFP with mCherry, the FastCloning method (Li et al., 2011) was performed using the following primers: EGFP-ATG-For, EGFP-AGG-Rev, out_EGFP-CAT-Rev and out_EGFP-AAG-For (Table S1).

The protoplasts of strain #58 were transformed with a mixture of pCc1003 (*trp1*^+^) and p*cag1*-mCherry, as described previously (Binninger et al., 1987). The trp^+^ transformants were recovered on minimal medium to purify the transformed cells. The purified trp^+^ transformants were crossed with a tester strain F_1_#2 in MYG slant media to allow fruiting. The transformants that exhibited dikaryotic normal fruiting were selected as strains with functional mCherry-tagged Cag1. Strain #58′Cag1-mCherry#15 was used in further experiments. Strain #58′Cag1-EGFP#1 was constructed in a similar manner. Strain #8 was also transformed with p*Cc.tupA*-EGFP or p*Cc.tupA*-mCherry.

### Microscopy

To observe the nuclei of vegetative hyphae, a small agar cube containing mycelium was inoculated on one side of a chamber, which was banked with two L-shaped glass rods on a coverslip (55×24 mm), sealed with minimal medium containing 2% agar, and poured with 400 µl of liquid minimal medium. After incubating for 2 or 3 days, the mycelium was attached to the coverslip by removing the liquid medium. After inverting the coverslip, the mycelium was observed using a fluorescence microscope.

To observe the pileus tissue and basidial cells, the primordial pileus was sectioned at a thickness 30–40 µm using a vibratome (Lancer Vibratome Series 1000, The Vibratome Company, USA). The sections were mounted on a glass slide with distilled water and covered with a coverslip before observation using a fluorescence microscope. Bright field and fluorescent images were captured with a BX51 fluorescence microscope (Olympus, Tokyo) equipped with a DP70 digital camera. Image processing was performed using Olympus DP manager and Adobe Photoshop Elements 10.

### Yeast two-hybrid assay

Yeast two-hybrid analyses were conducted using the Matchmaker Gold Yeast Two-hybrid System (Clontech Laboratories, Inc.). Strain Y2HGold was transformed with pGBKT7 (marked with *TRP1*) where cDNA from *cag1*, *Cc.tupA* or a part of *Cc.cyc8* was inserted in-frame. Strain Y187 was transformed with pGADT7 AD (marked with *LEU2*) where cDNA from *cag1*, *Cc.tupA* or the part of *Cc.cyc8* was also inserted in-frame. Expression of DBD and AD fusion proteins was confirmed by western blotting using anti-c-Myc antibody and anti-HA antibody, respectively. The transformants that expressed the fusion proteins were mated with each other to examine their interaction. Truncated versions of Cag1 and Cc.TupA were produced by digesting with appropriate restriction enzymes to delete the N-terminal or C-terminal region. The cloned cDNA samples were also used as standard templates for qRT-PCR.
